# Modelling the association between psychosocial work factors and risky driving: mediating roles of burnout and engagement

**DOI:** 10.3389/fpubh.2026.1766587

**Published:** 2026-04-10

**Authors:** Mustapha Amoadu, Edward Wilson Ansah

**Affiliations:** 1 Biomedical and Clinical Research Centre, University of Cape Coast, Cape Coast, Ghana; 2 Department of Health, Physical Education and Recreation, University of Cape Coast, Cape Coast, Ghana

**Keywords:** Ghana, heavy goods drivers, job burnout, job demands, job engagement, job resources, PLS-SEM, risky driving behaviours

## Abstract

**Introduction:**

Drivers of heavy goods vehicles (HGVs) face significant psychosocial work challenges that increase their job burnout and risky driving behaviours, affecting drivers’ well-being and on-the-road safety. However, these psychosocial work challenges have received limited research attention in developing countries. This study explored the relationships among job demands, job resources, job burnout, job engagement, and risky driving behaviours in Ghanaian HGV drivers.

**Method:**

This cross-sectional survey collected data from 1,575 HGV drivers (truck and tanker drivers) in Tema, Ghana. Data were collected using a validated questionnaire and analyzed with partial least squares structural equation modelling (PLS-SEM)—SmartPLS.

**Results:**

High job demands is associated with high job burnout and risky driving behaviours among HGV drivers in Ghana, while job burnout positively correlates with the occurrence of risky driving behaviour. High job resources are associated with low job burnout and high job engagement among the drivers. However, high job resources did not have a statistically significant association with low risky driving behaviour among the drivers. Mediation analyses revealed that job burnout partially mediated the relationships between job demands and risky driving behaviour, and job resources and risky driving behaviour. Moreover, job engagement mediated the relationships between job resources and risky driving behaviour. Moreover, job resources significantly buffered the effects of job demands on job burnout. Job resources further moderated the influence of job burnout on risky driving behaviours.

**Conclusion:**

This study suggests that psychosocial work factors strongly influenced job burnout and associated risky driving behaviours among Ghanaian HGV drivers. Efforts to balance job demands and enhance support systems are critical to improving the health, safety, and well-being of these drivers and ultimately reducing road accidents in the country.

## Introduction

Heavy goods vehicle (HGV) drivers are indispensable to the global supply chain sector, facilitating the movement of goods across countries and regions. However, the occupational challenges these drivers face, and the associated road safety consequences, remain a major public health concern (1, 2). HGV drivers work long hours with irregular schedules, get minimal rest, are poorly paid, and have limited access to healthcare (1). These precarious working conditions not only threaten drivers’ health and well-being but also heighten the risk of road traffic crashes (RTCs), posing a serious danger to public safety (2, 3). The World Health Organisation’s (WHO) global estimates indicate that RTCs claim approximately 1.19 million lives annually due to human errors, often influenced by fatigue, stress, and other occupational factors (4).

In Africa, the RTC crisis is alarming, with approximately 26.6 deaths per 100,000 population (5). In Ghana, RTCs account for 24.9 deaths per 100,000 people, which remains substantially higher than the global average of 18.2 per 100,000 (6). The RTC figures show that Ghana shares a similar high burden of RTC-related deaths with the continent of Africa. However, the regional and local RTC-related deaths per 100,000 substantially exceed the global average, indicating a disproportionate impact within the region. These patterns of RTC-related deaths in Africa show the urgent need to strengthen road infrastructure, road safety policies, enforcement mechanisms, and system-level interventions across the continent.

HGV drivers contribute significantly to these statistics because of the inherent risks associated with their work. Crashes involving HGVs tend to be more severe than those involving smaller vehicles because of the high mass of HGVs (7). These crashes often result in serious injuries and/or fatalities, particularly among occupants of smaller vehicles such as cars and motorcycles (6). Notably, over 80% of fatalities in secondary vehicles are linked to collisions involving HGVs (8). On average, in Ghana, when a heavy vehicle driver dies in a RTC, three other road users also lose their lives (9).

In developed countries, significant strides have been made in integrating contemporary occupational health and safety (OHS) practices into the road transport industry (10). These efforts have facilitated evidence-based interventions that enhance the safety of road users (11). For example, Europe and other high-income regions have substantially reduced occupational traffic crashes through the implementation of robust regulations, policies, and OHS research tailored to the road transport sector (10, 12). However, developing countries continue to face deteriorating conditions in their road transport sector (13). In Ghana, OHS practices remain underdeveloped, with the framework still in its infancy (14). Consequently, road transport accidents, associated injuries, and fatalities are not only common but also increasingly challenging to address (14).

While efforts to reduce risky driving behaviours such as speeding or substance use are important, they often overlook the broader work environment in which HGV drivers operate (14). In Ghana, long driving hours, lack of job security, inadequate support from vehicle owners, poor remuneration, and limited rest stop facilities contribute to high stress among these drivers (13). These challenges harm drivers’ health and well-being and also increase the risk of errors and RTCs (12). Additionally, the poor work environment hinders progress towards the Sustainable Development Goals (SDGs), particularly Goal 8.5, which promotes decent, safe, and healthy workplaces, and Goal 3.6, aimed at halving RTCs by 2030.

A recent review identified limited evidence from the African region on psychosocial work factors such as psychological demands and social support on risky driving and RTCs (3). The review further reported that psychosocial work factors among hazardous transport drivers in low- and middle-income countries (LMICs) remained underexplored, with few studies (15, 16) addressing these critical occupational concerns. This limited research attention has contributed to a narrow understanding of the underlying causes of risky driving in LMICs. Moreover, despite the critical role of HGV drivers in national and regional economies, there is limited research on their psychosocial work environment in Ghana. Consequently, current road safety interventions tend to focus predominantly on individual risky driving behaviours, while overlooking the broader systemic and workplace factors that contribute to these on-the-road behaviours (17). This gap persists partly due to the dominant behavioural framing of road safety in Ghana, which tends to individualise responsibilities for the occurrence of RTCs (13). As a result, systemic work-related stressors remain underexplored, limiting the development of holistic interventions that address the root causes of risky driving. Bridging this gap is essential for advancing theory and practice in road safety within sub-Saharan Africa (13, 17).

This study sought to model the relationships among psychosocial work factors, job burnout, job engagement, and driver behaviour among HGV drivers in Ghana. In this study, psychological demands were selected as the key job demands because HGV drivers in Ghana often face long working hours, tight schedules, creating mental fatigue, which could increase stress and associated risky driving behaviours (13). Social support from supervisors and co-workers was chosen as a job resources because it is a practical and accessible form of support in the sector. These variables reflect common and relevant aspects of the drivers’ daily work experiences that influence their well-being and road safety. The findings provide knowledge useful for designing interventions that address the root cause of risky driving, improving driver well-being, and road safety. Thus, these findings have the potential to inform occupational health and transport policies by demonstrating the need to integrate psychosocial risk management into road safety strategies. The findings could guide employers, regulatory institutions, such as the National Road Safety Authority, and other policymakers in developing context-specific interventions that prioritise supportive work environments alongside behavioural road safety measures.

## Theoretical model

The job demand-resource (JD-R) model (18) underpins this study. The main argument of the JD-R theory is that job demands lead to strain and negative outcomes (the health erosion path), while job resources promote motivation and positive outcomes (the motivation path), with both processes influencing overall job performance and well-being of the worker. The model highlights two pathways: the health erosion hypothesis, where excessive job demands without adequate job resources leads to job burnout, compromising health, and likely increase in risky driving behaviours. The other is the motivational hypothesis, where job resources (in this study, including support from supervisors and co-workers) enhance job engagement, fostering motivation and reducing unsafe driving behaviours. In this study, job demands and job resources serve as predictors, with job burnout and job engagement acting as mediators, explaining the mechanisms through which these factors impact risky driving behaviours. The JD-R model provides a practical framework for understanding the dual influence of stress-inducing demands and protective resources from the organisational and worker perspectives.

This study extends the JD-R model by introducing job resources, specifically, social support from supervisors and co-workers as moderating variables in two key pathways: the relationship between job demands and job burnout and between job burnout and risky driving behaviours. Unlike the traditional JD-R applications that primarily position job resources as direct predictors or mediators of job engagement (18), this study conceptualises downstream job resources (e.g., interpersonal support) as buffering mechanisms that mitigate the adverse effects of job demands, job burnout, and risky driving behaviours. Authors argue that by applying this extended framework in the context of HGV drivers in Ghana, the study provides novel insights into how interpersonal support could dampen the health erosion process and reduce the likelihood of unsafe driving behaviours in these high-risk occupational settings. In a resource-limited setting and within a largely informal occupational group, such as long-distance HGV drivers in Ghana, downstream job resources, such as supervisors’ and co-drivers’ support, are particularly crucial. These interpersonal forms of support often substitute for more structured organisational resources such as formal employee assistance programmes, occupational health services, and robust enforcement of labour protections, which are typically absent or poorly implemented in such contexts. The extension of the JD-R model to include social support as a moderator may help explain why these lower-level job resources are effective in reducing the impact of job stress on risky driving behaviours, offering a more context-sensitive understanding of how drivers could cope with high job demands and job burnout.

## Hypotheses development

### Health erosion hypothesis

Within the JD-R framework, job demand is theorised as a trigger of the health impairment process, whereby sustaining the exposure to excessive workload, time pressures, and psychological strain that progressively deplete energy reserves of the workers (19). This depletion compromises cognitive functioning, induces emotional exhaustion, and undermines drivers’ capacity to make accurate judgements, ultimately heightening the risk of unsafe driving behaviours (20–22). Job demands, such as excessive working hours, high workload, and time pressures, drains the physical and mental resources of these drivers, leading to job burnout (18, 23). Prolonged exposure to these demands exacerbates stress and exhaustion, making drivers very vulnerable to job burnout (24). Among commercial drivers, studies revealed that high job demand significantly elevates job burnout levels (25, 26). This burnout contributes to risky driving behaviours, as fatigue and stress impair decision-making and weaken adherence to safety protocols on the road (27, 28). Professional drivers facing time pressures or heavy workloads are more likely to engage in unsafe practices, such as excessive speeding and or ignoring traffic rules (26). In contrast, high job resources mitigate job burnout by fostering resilience and providing support for workers (26). Job resources, such as supportive supervision and flexible schedules, reduce burnout and help drivers cope effectively with demands (23, 25). For drivers, these job resources strengthen physical and psychological resilience, thereby lowering job burnout (26). A key concern is that burnout impairs cognitive functioning and decision-making, which further increase risky driving behaviours (28). Research among drivers has shown that job burnout is strongly associated with unsafe driving practices (21).

Evidence further shows that job demands and job resources affect driving performance through job burnout (10). This reflects the JD-R framework’s position that job burnout is not merely a downstream consequence of high job demands but a core mediating mechanism that link work conditions to behavioural outcomes such as risky driving (21, 28). Specifically, job burnout compromises essential psychological capacities, including self-regulation, vigilance, and risk appraisal, which are critical for safe driving performance (29, 30). Through this lens, job burnout explains how high job demand translates into impaired decision-making and associated high risky driving behaviours. Equally, job resources influence risky driving behaviours indirectly by mitigating job burnout (28). Supportive interpersonal job resources buffer the psychological impact of stressors, thereby preserving drivers’ mental energy and reducing their susceptibility to job burnout-induced lapses while on the road (31, 32). Hence, job burnout serves as a converging pathway through which both the presence of job demands and the absence or inadequacy of job resources affect safety-critical behaviours. Therefore, we hypothesised that:

*H1*: Job demand has a positive and significant relationship with job burnout.

*H2*: Job burnout has a positive and significant relationship with risky driving behaviours.

*H3*: Job resource has a negative and significant relationship with job burnout.

*H4*: Job demand has a positive and significant association with risky driving behaviours.

*H5*: Job burnout significantly mediates the association between job demand and risky driving behaviours.

H_6_: Job burnout significantly mediates the association between job resources and risky driving behaviours.

### Motivation hypothesis

In the JD-R model, job resources activate the motivational process by enhancing individuals’ capacity to remain committed, focused, and psychologically resilient in the face of occupational challenges (19). In the transport sector, supportive interactions with supervisors and peers not only bolster job engagement but also buffer the negative effects of high job demands, thereby promoting adherence to safe driving behaviours and reducing the propensity for risk-taking and accidents (25, 28, 33). Dollard and Bakker (34) found that access to job resources positively influenced energy and dedication at work. While the link between job resources and job engagement among drivers is underexplored, existing studies indicate a positive relationship between these variables (35, 36). Job resources serve as protective factors against unsafe driving behaviours by providing drivers with motivation and support to follow safety standards while on the road (21). The connection between job resources and driving performance is well documented (12, 21). Job burnout diminishes job engagement by draining the emotional and physical resources (37), leading to disengagement and decreased motivation among workers (19, 37). However, this relationship is extensively examined among professional drivers.

Furthermore, job resources influence organisational and employee outcomes through job engagement (19, 38). Within the motivational pathway of the JD-R model, job resources do not merely reduce stress, but more importantly, they stimulate employees’ intrinsic motivation, leading to greater job engagement (38–41). Job engagement reflects a psychological state in which workers are energised, focused, and committed to their tasks (35). This state is particularly critical for drivers whose performance depends heavily on sustained attention, emotional regulation, and compliance with safety norms. As a mediating variable, job engagement explains how job resources translate into safer on-the-road behaviours (35). Drivers with high job engagement are more likely to internalise safety protocols, remain vigilant, and resist distractions or shortcuts that could result in risky driving and associated consequences (35). Empirical evidence in transport sectors affirms these pathways: access to job resources enhances job engagement, which, in turn, reduces counterproductive or hazardous behaviours (35, 42, 43). Thus, job engagement acts as the motivational pathway linking job resources to behavioural outcomes, demonstrating that the value of job resources lies not only in buffering stress but also in fostering proactive energy and focus, which underpin safe and responsible driving. Hence, these hypotheses:

*H7*: Job resource has a positive and significant association with job engagement.

*H8*: Job engagement has a negative and significant association with risky driving behaviours.

*H9*: Job resource has a negative and significant association with risky driving behaviours.

*H10*: Job engagement significantly mediates the association between job resources and risky driving behaviours.

*H11*: Job burnout has a negative and significant association with job engagement.

### Buffering effect of job resources

Demerouti et al. (18) demonstrated that job resources mitigate the adverse effects of high job demands on employee well-being. This buffering effect protects employees from experiencing excessive job burnout by providing the necessary job resources to cope with high job demands effectively (44). The buffering effect of job resources means that in a high job resource work setting, the effect of high job demands on job burnout, or the effect of job burnout on job performance, could be reduced significantly. The moderating role of job resources in the health erosion pathway has been explored in the general working population (44, 45) but not among HGV drivers in Africa. A review shows that previous studies have consistently applied the JD-R model in a more conventional manner (19), often without exploring alternative roles of job resources in settings with limited infrastructure and institutional support. This consistency, while valuable, points to an opportunity to expand and test the model’s boundaries. Hence, we hypothesised that:

*H12*: Job resource significantly moderates the association between job demand and job burnout.

*H13*: Job resource significantly moderates the relationship between job burnout and risky driving behaviours.

## Methods

### Designs and population

This cross-sectional survey involved 1,575 HGV drivers in Ghana, consisting of 910 truck (haulage) drivers and 665 tanker drivers. The sample size represented 26.6% of the estimated target population of 5,312 HGV drivers. This population included 3,240 haulage drivers operating at the Tema Port truck terminal and 2,072 tanker drivers working at seven bulk storage terminals in the same area. The sample size was informed by the “10-times rule” in partial least squares structural equation modelling (PLS-SEM), which recommends that the number of cases should be at least 10 times the maximum number of structural paths pointing to any single latent construct in the model (46). In this study, the most complex construct, risky driving behaviours, had four predictor variables, suggesting a minimum requirement of 40 cases. The achieved sample size, therefore, exceeded this threshold substantially, providing strong statistical power and model stability. Moreover, the large sample size was suitable for testing indirect and interaction effects, which are critical demands in statistical precision.

In this study, long-distance driving refers to drivers who travel at least 140 km per trip. The truck drivers are registered members of the Ghana Haulage Truck Drivers Association, and they operate from the Tema Port truck terminal. Similarly, the tanker drivers are members of the National Petroleum Tanker Drivers Union and the Liquefied Petroleum Gas Tanker Drivers Union. All the drivers included in the study were full-time workers and held professional licenses issued by the Driver and Vehicle Licensing Authority (DVLA) of Ghana.

## Measures

The drivers provided information about their age, highest educational attainment, marital status, years of work experience as professional truck/tanker drivers, daily driving hours, and weekly working days. The summary is presented in Table 1.

**Table 1:** Details of measures.

Measures	Source and example of item	No. of items	Response set	Cronbach’s alpha (*α*)
Job demands	Job content questionnaire (JCQ) (31) For example, “My job requires excessive work.”	6 items	Strongly disagree (SD) to strongly agree (SA).	0.874
Supervisor support	JCQ (31) For example, “My vehicle owner/station master is concerned about the welfare of those who work under him or her.”	4 items	SD to SA	0.897
Co-worker support	JCQ (31) For example, “My station master/car owner is successful in getting you to work together.”	4 items	SD to SA	0.813
Job burnout	Copenhagen Psychosocial Questionnaire (COPSOQ II) (32) For example, “How often have you been physically exhausted.”	4 items	“Not at all” to “All the time”	0.931
Job engagement	Copenhagen Psychosocial Questionnaire (COPSOQ II) (32) For example, “I am immersed in my work.”	3 items	“Hardly ever” to “Nearly all the time”	0.838
Risky driving behaviour	Driver Behaviour Questionnaire (DBQ) (33). For example, “Drove above your speed limit.”	5 items	“Hardly ever” to “Nearly all the time”	0.839

### Procedures and ethics

Participants were recruited on-site at the Tema Port truck terminal and the seven bulk storage terminals with the support of union executives and company administrators. Drivers who were present and waiting for their next assignments were approached and invited to participate in the study using a convenience sampling approach. Data collection was conducted through face-to-face survey interviews immediately after informed consent was obtained. Therefore, reminders were not required. Access to participants was facilitated through supervisors and union leaders, who granted permission for engagement but were not involved in the data collection process and did not influence participation or responses. Participation was entirely voluntary, and confidentiality was assured throughout the study. Drivers waiting for their next load were sampled for the study using a convenience sampling method. The questionnaire was translated into Twi, a widely spoken local language, and back-translated into English by a language expert from the University of Cape Coast to ensure accuracy. Data collection was conducted with the help of eight trained field assistants through survey interviews that lasted 25–45 min. The survey interviews took place over 3 months, from June to August 2023. Drivers were informed of their right to withdraw from the study at any time and were assured of their confidentiality. For drivers who could not read or understand the English language, field assistants explained the consent form before obtaining their written consent. No financial or material incentives were provided to participants for participation in this study. This study received ethical approval from the Institutional Review Board of the University of Cape Coast, Ghana (ID: UCCIRB/CES/2022/82). Prior to data collection, a pre-test involving 91 HGV drivers in the Takoradi Metropolis was conducted to evaluate the psychometric properties of the survey.

### Analytical procedures

Hypotheses (paths) in the proposed model were tested using PLS-SEM in Smart PLS version 4.1.0.9. The procedures proposed by Hair et al. (47) in analysing path models were followed. PLS-SEM was chosen over covariance-based SEM because the primary objective of this study was prediction and theory extension, rather than theory confirmation (47). PLS-SEM is also particularly appropriate for complex models involving multiple mediators and moderators, as it handles latent variables with non-normal data distributions, it accommodates larger models, and it places fewer restrictions on data normality and measurement scales (47). Given the applied and exploratory nature of this study, aiming at understanding the pathways linking psychosocial work factors to risky driving behaviours among Ghanaian HGV drivers, PLS-SEM provided the flexibility and statistical power necessary for robust path estimation. Moreover, the technique is well-suited for assessing both reflective measurement models and structural relationships simultaneously, enabling comprehensive evaluation of direct, indirect (mediation), and moderating effects in a single analytical.

### Model specification and assessment of outer model

**Figure 1:**
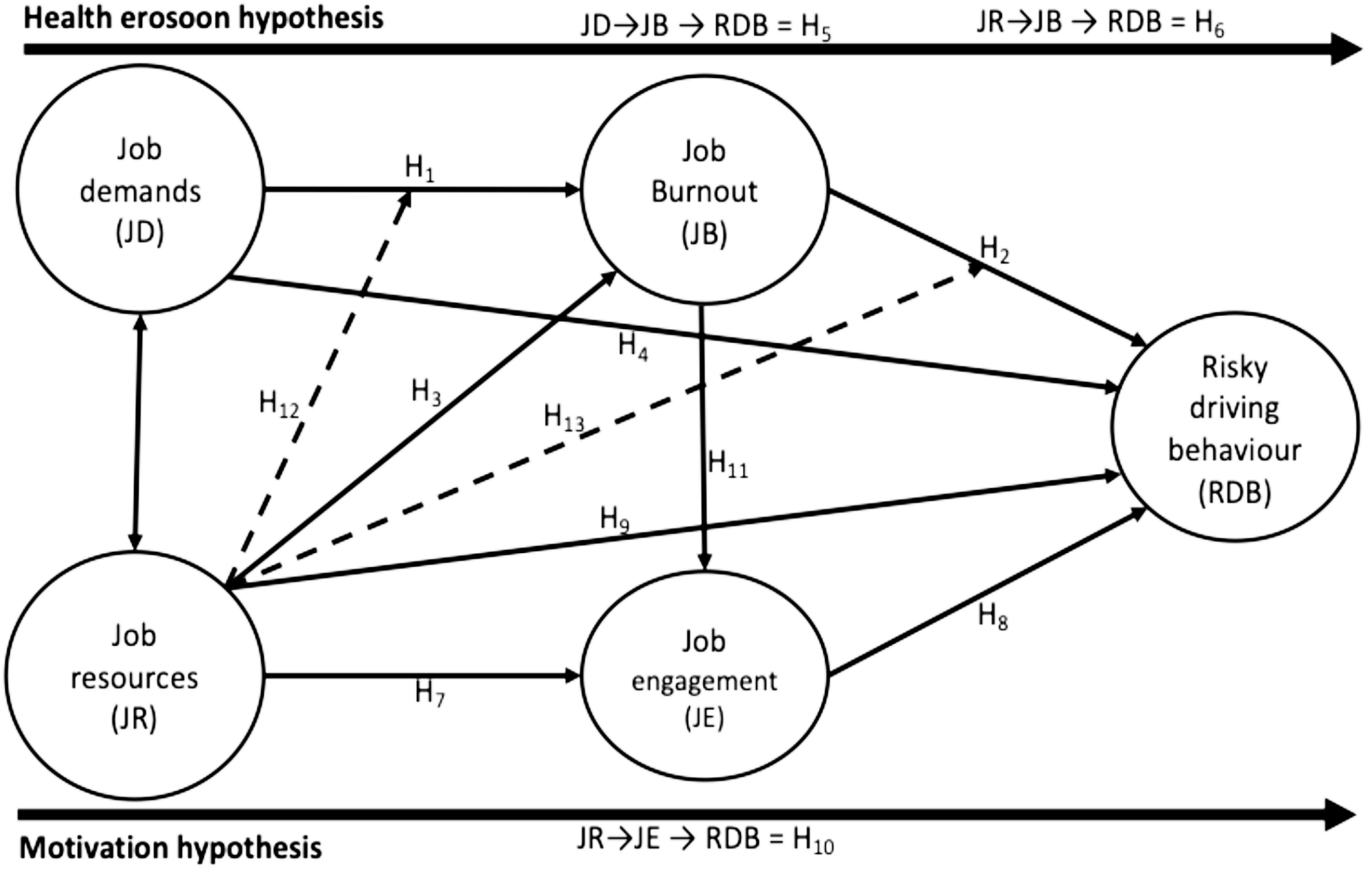
Structural model based on the JD-R model.

The predictors (job demand and job resource), mediators (job burnout and job engagement), and the outcome variable (risky driving behaviours) were quantitative latent variables measured with quantitative indicators, and they modelled reflectively (see Figure 1). Composite reliability (CR) with acceptable values ≥0.70 (47) was used to establish the internal consistency of the constructs in the path models. Average variance extracted (AVE) values ≥0.50 (47) were used to assess convergent validity. To assess the discriminant validity of the constructs, the criteria of Fornell and Larcker (48) and heterotrait-monotrait ratio of correlation (HTMT) values (<0.90) were applied (see Tables 2, 3). Indicators with outer loadings <0.70 were deleted, and the analysis continued until the desired outer loading (≥0.70) was achieved. This led to the deletion of two items measuring social support (job resource 5 and job resource 8), which were loading <0.70, but this did not negatively affect the conceptual coverage of the construct. The outer loadings, CR, Cronbach’s alpha (*α*), and AVE of constructs in the path model are presented in Table 4.

**Table 2:** HTMT ratio of correlations.

Constructs	JB	RDB	JD	JE	JR
JB	_				
RDB	0.725	_			
JD	0.787	0.753	_		
JE	0.654	0.888	0.671	_	
JR	0.668	0.796	0.689	0.841	_

JB, job demand; RDB, risky driving behaviour; JD, job demand; JE, job engagement; JR, job resource.

**Table 3:** Fornell and Larcker (1981) criterion.

Constructs	JB	RDB	JD	JE	JR
JB	0.913				
RDB	0.690	0.903			
JD	0.759	0.743	0.780		
JE	−0.617	−0.838	−0.664	0.941	
JR	−0.633	−0.764	−0.676	0.804	0.840

JB, job demand; RDB, risky driving behaviour; JD, job demand; JE, job engagement; JR, job resource.

**Table 4:** Outer loading, *α*, CR and AVE of constructs.

Latent constructs	Outer loadings				
	JB	RDB	JD	JE	JR
JB (*α* = 0.933, CR = 0.937, AVE = 0.834)					
JB_1	0.945				
JB_2	0.925				
JB_3	0.937				
JB_4	0.844				
RDB (*α* = 0.943, CR = 0.949, AVE = 0.816)					
DBQ_1		0.885			
DBQ_2		0.954			
DBQ_3		0.950			
DBQ_4		0.916			
DBQ_5		0.803			
JD (*α* = 0.875, CR = 0.907, AVE = 0.609)					
JD_1			0.831		
JD_2			0.836		
JD_3			0.752		
JD_4			0.709		
JD_5			0.745		
JD_6			0.800		
JE (*α* = 0.935, CR = 0.941, AVE = 0.885)					
JE_1				0.932	
JE_2				0.958	
JE_3				0.932	
JR (*α* = 0.917, CR = 0.937, AVE = 0.706)					
JR_1					0.875
JR_2					0.907
JR_3					0.852
JR_4					0.765
JR_6					0.820
JR_7					0.814

JB, job demand; RDB, risky driving behaviour; JD, job demand; JE, job engagement; JR, job resource.

#### Assessment of inner model

The assessment of the inner model started with the assessment of the multicollinearity. The Fornell and Larcker’s criterion was used; it was found that issues of multicollinearity did not exist in the path model (see Table 3). This was further confirmed using the variance inflation factor (VIF) (see Table 5), which was between the acceptable range (VIF > 0.10 and <5) (47, 48). Standardised root mean square residual (SRMR) was then used to assess the model fit with a criterion of ≤0.10 (49). The SRMR of 0.10 of the inner model was acceptable.

**Table 5:** VIF and *f*^2^ of the path coefficients.

Paths	VIF	*f* ^2^	SD	T statistics	*p*-values
Job burnout → job engagement	2.892	0.059	0.010	6.101	0.000
Job burnout → RDB	1.667	0.056	0.012	4.754	0.000
Job demand → job burnout	2.101	0.383	0.032	12.067	0.000
Job demand → RDB	3.100	0.113	0.020	5.603	0.000
Job engagement → RDB	3.106	0.385	0.039	9.783	0.000
Job resource → job burnout	1.998	0.090	0.016	5.731	0.000
Job resource → job engagement	3.784	0.862	0.076	11.226	0.000
Job resource → RDB	1.667	0.003	0.003	0.722	0.471
Job resource × job demand → job burnout	1.144	0.031	0.008	3.541	0.000
Job resource × job burnout → RDB	1.529	0.046	0.012	3.661	0.000

SD, standard deviation; VIF, variance inflation factor; RDB, risky driving behaviour.

**Figure 2:**
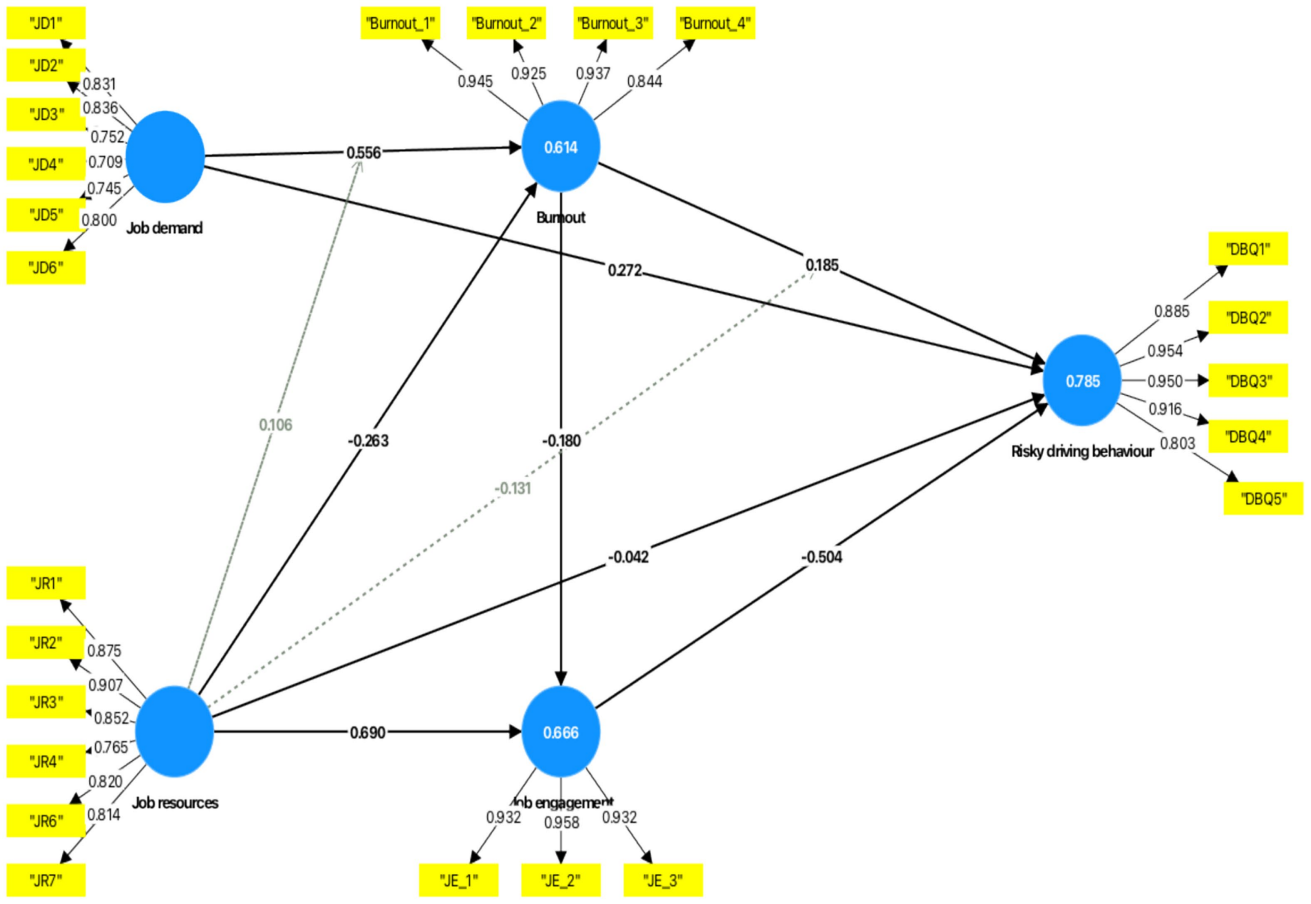
Path coefficients, *p*-values, and outer weights of the path model.

The adjusted *R*^2^ (*R*^2^_adj_) was used to determine the predictive ability of the model. Job demand, job resource, job burnout and job engagement explained 78.5% of variance in risky driving behaviours (*R*^2^_adj_ = 0.785). Job demand and job resource explained 61.4% of variance in job burnout (*R*^2^_adj_ = 0.614), and job resource and job burnout explained 66.6% of variance in job engagement (*R*^2^_adj_ = 0.666) (see the details in Figure 2). Cross-validated redundancy (*Q*^2^) was used to assess the predictive relevance of the inner model with the Stone-Geisser criterion of *Q*^2^ > 0 (47). The three endogenous constructs, job burnout, job engagement, and risky driving behaviours had *Q*^2^ values of 0.608, 0.664, and 0.691, respectively, and the higher the value is from zero, the better the predictive relevance of the construct. Finally, the bootstrapping process (5,000 resamples) assessed the path coefficients of the model, its significance (see Figure 2) and effect size (see Table 5) using Cohen’s *f^2^*. A path is significant if the *t*-value is >1.96 at *p* < 0.05. Cohen’s *f*^2^ of 0.02, 0.15, and 0.35 imply small, moderate, and large practical effect sizes, respectively (49). Bootstrapping is a non-parametric resampling technique that repeatedly draws subsamples (with replacement) from the original dataset to generate empirical standard errors, *t*-values, and confidence intervals. This approach does not assume normal data distribution and is particularly appropriate for complex mediation and moderation models.

#### Evaluation of mediation and moderation models

To evaluate the mediation role of job burnout and job engagement, the direct paths need to be significant to establish the basis for assessing the mediation as partial or full mediation (47). In full mediation, the direct path is no more significant in the presence of the mediator(s). In partial mediation, the direct paths are still significant but reduced when the mediators are present in the model (47). The moderating roles of job resources were examined using the two-stage approach (47). The significance of the interaction effects (H_12_ and H_13_) was assessed using the bootstrapping process, which is supported when the *t*-value is >1.96 at *p* < 0.05. The *f*^2^ of the interaction effect was evaluated using the criterion of 0.005, 0.01, and 0.25 as small, moderate, and large effects, respectively (47). Slop plots were then presented.

## Results

### Socio-demographic characteristics of the HGV drivers

The study included 1,575 drivers, predominantly males (94.7%), with a mean age of 39.2 years and an average HGV driving experience of 13.2 years. Educational levels varied, with 39.7% having basic education and 11.4% attaining tertiary education. Half of these drivers were single (50.2%), and the average monthly salary was $92.13, about 1,500 Ghana cedis (at the time of data collection).

#### Testing hypotheses

The results supported Hypothesis 1, showing that job demand had a positive association with job burnout (*r* = 0.556, *t* = 29.609, *p* < 0.001), and Hypothesis 2, which proposed that job burnout is positively associated with risky driving behaviours (*r* = 0.185, *t* = 10.333, *p* < 0.001). Hypothesis 3 was also confirmed, as job resources had a negative association with job burnout (*r* = −0.263, *t* = 13.018, *p* < 0.001). Hypothesis 4 was supported, with job demand showing a positive association with risky driving behaviours (*r* = 0.272, *t* = 12.093, *p* < 0.001). Consistent with the health erosion hypothesis (Hypothesis 5), job burnout partially mediated the relationship between job demand and risky driving behaviours, as both the direct path (job demand → risky driving behaviours) and the indirect path (job demand → job burnout → risky driving behaviours) were statistically significant. This indicates that job demand influences risky driving behaviours both directly and indirectly through high job burnout. The mediation analysis supported Hypothesis 6, indicating that job burnout significantly mediated the relationship between job resources and risky driving behaviours. Although the indirect effect (job resource → job burnout → risky driving behaviours) was statistically significant, the direct path from job resource to risky driving behaviours became non-significant when job burnout was included in the model. This pattern demonstrates full mediation, suggesting that job resources reduce risky driving behaviours primarily by lowering levels of job burnout rather than exerting a direct behavioural influence (see Table 6).

**Table 6:** Mediation effects in the path model.

Paths	Path co-efficient (*r*)	SD	T statistics	*p*-values (*p*)
Total effects				
Job burnout → RDB	0.126	0.016	7.820	0.000
Job demand → job burnout	0.611	0.017	35.587	0.000
Job demand → RDB	0.302	0.017	17.817	0.000
Job engagement → RDB	−0.518	0.022	23.479	0.000
Job resource → job burnout	−0.219	0.020	10.756	0.000
Job resource → job engagement	0.804	0.010	77.453	0.000
Job resource → RDB	−0.560	0.017	32.878	0.000
Total indirect effects				
Job demand → RDB	0.077	0.010	7.409	0.000
Job resource → RDB	−0.444	0.020	22.363	0.000
Specific indirect effects				
Job burnout → job burnout → RDB	0.077	0.010	7.409	0.000
Job resource → job engagement → RDB	−0.416	0.020	21.203	0.000
Job resource → job burnout → RDB	−0.028	0.004	6.615	0.000

SD, standard deviation; RDB, risky driving behaviour.

Hypotheses 7 and 8 were supported, as job resource had a positive association with job engagement (*r* = 0.690, *t* = 47.134, *p* < 0.001), and job engagement showed a negative association with risky driving behaviours (*r* = −0.504, *t* = 22.310, *p* < 0.001). Hypothesis 9, which posited a negative association between job resource and risky driving behaviours, was not supported (*r* = −0.042, *t* = 1.557, *p* = 0.120). Hypothesis 10 was supported, as job engagement significantly mediated the association between job resources and risky driving behaviours. The indirect effect (job resource → job engagement → risky driving behaviours) was statistically significant, while the direct effect of job resource on risky driving behaviours remained non-significant in the presence of job engagement. This indicates full mediation, meaning that job resources influence risky driving behaviours mainly by enhancing driver job engagement rather than through a direct pathway. Hypothesis 11 confirmed that job burnout had a negative association with job engagement (*r* = −0.180, *t* = 13.068, *p* < 0.001). All the mediation effects were partial, as both direct and indirect paths remained significant in the presence of the mediators (see Table 6).

**Figure 3:**
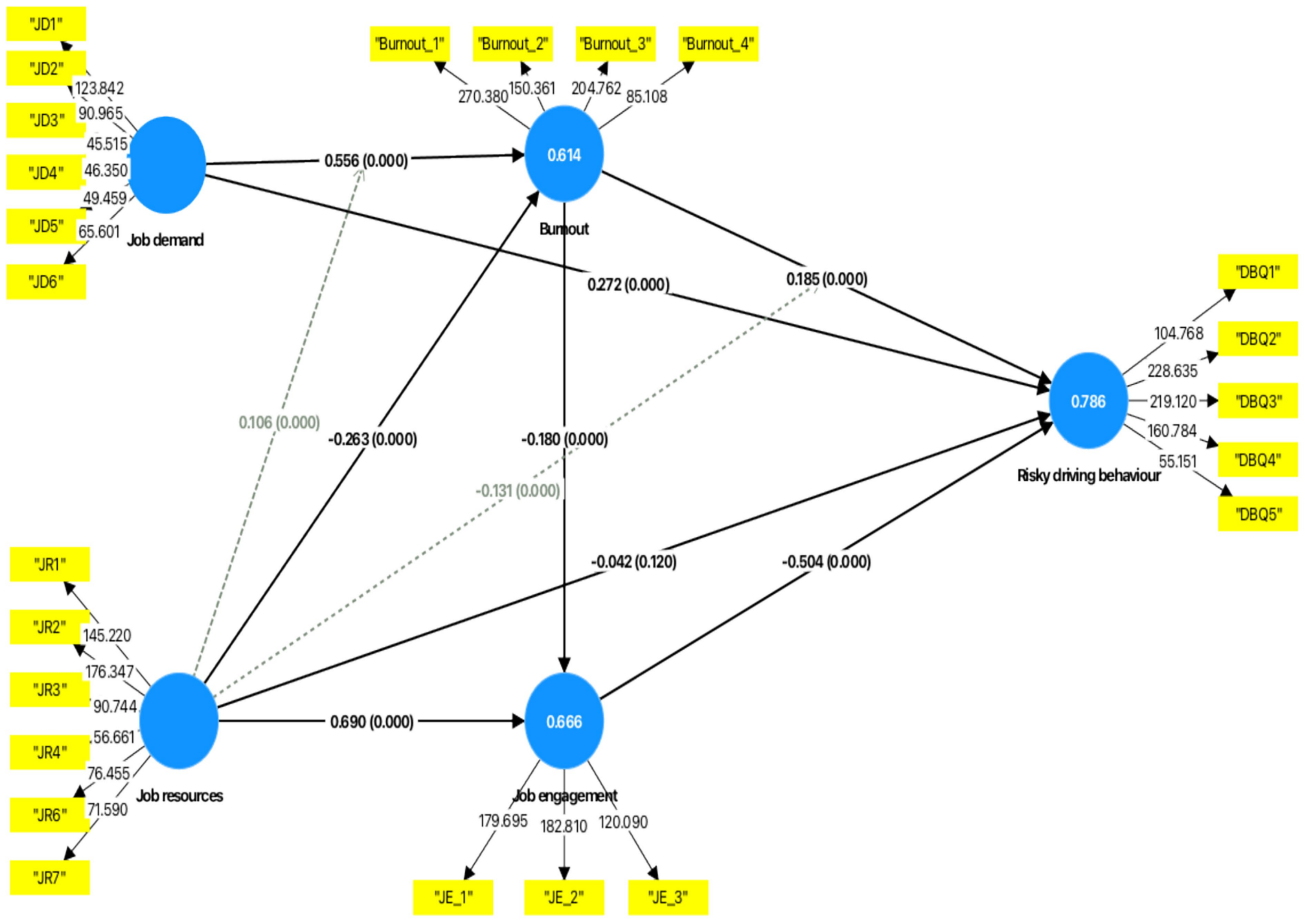
Path coefficients, *p*-values, and outer weights of the path model.

**Figure 4:**
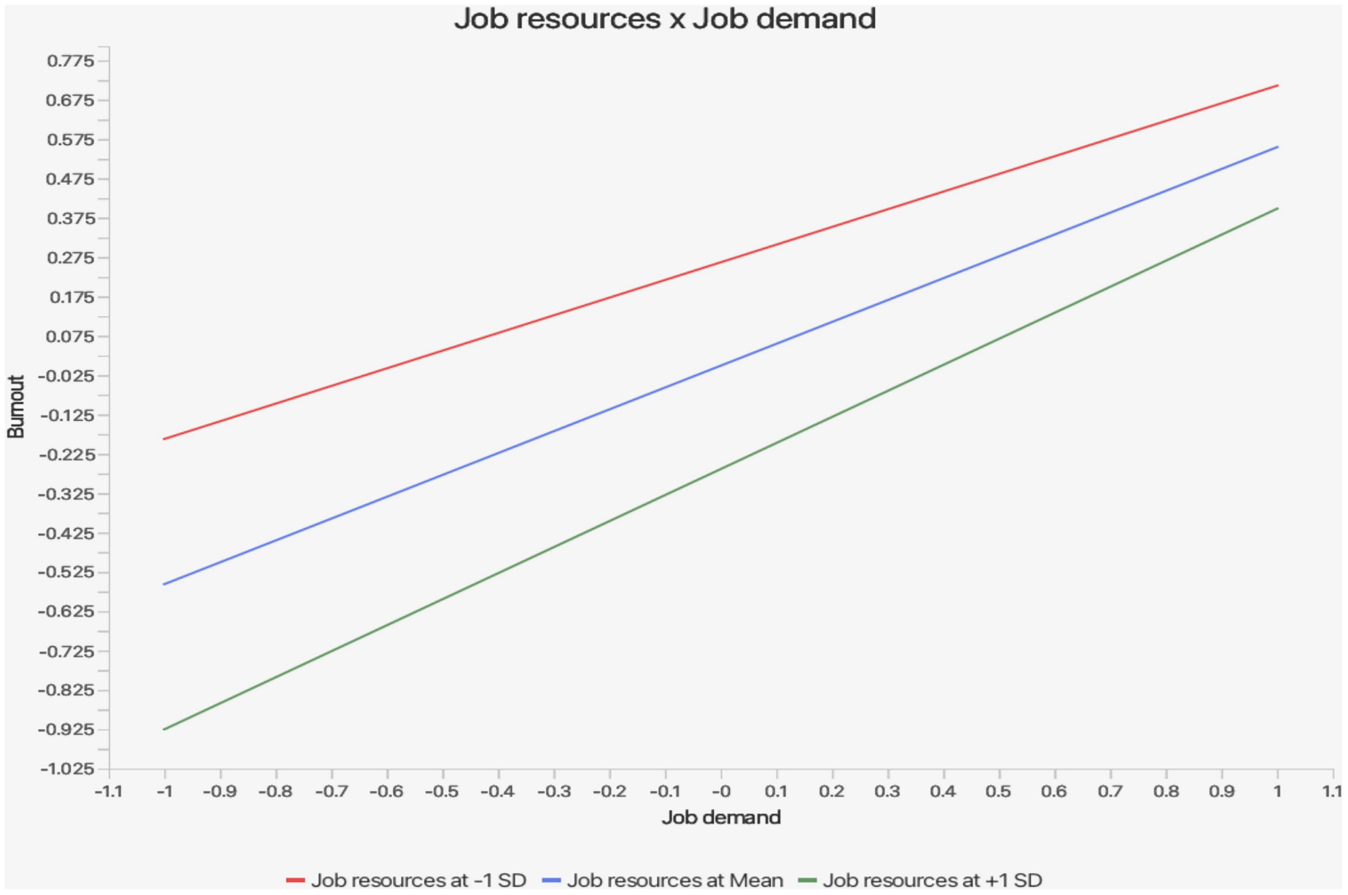
Slope plots for the moderating role of job resources on the path job burnout → risky driving behaviours.

**Figure 5:**
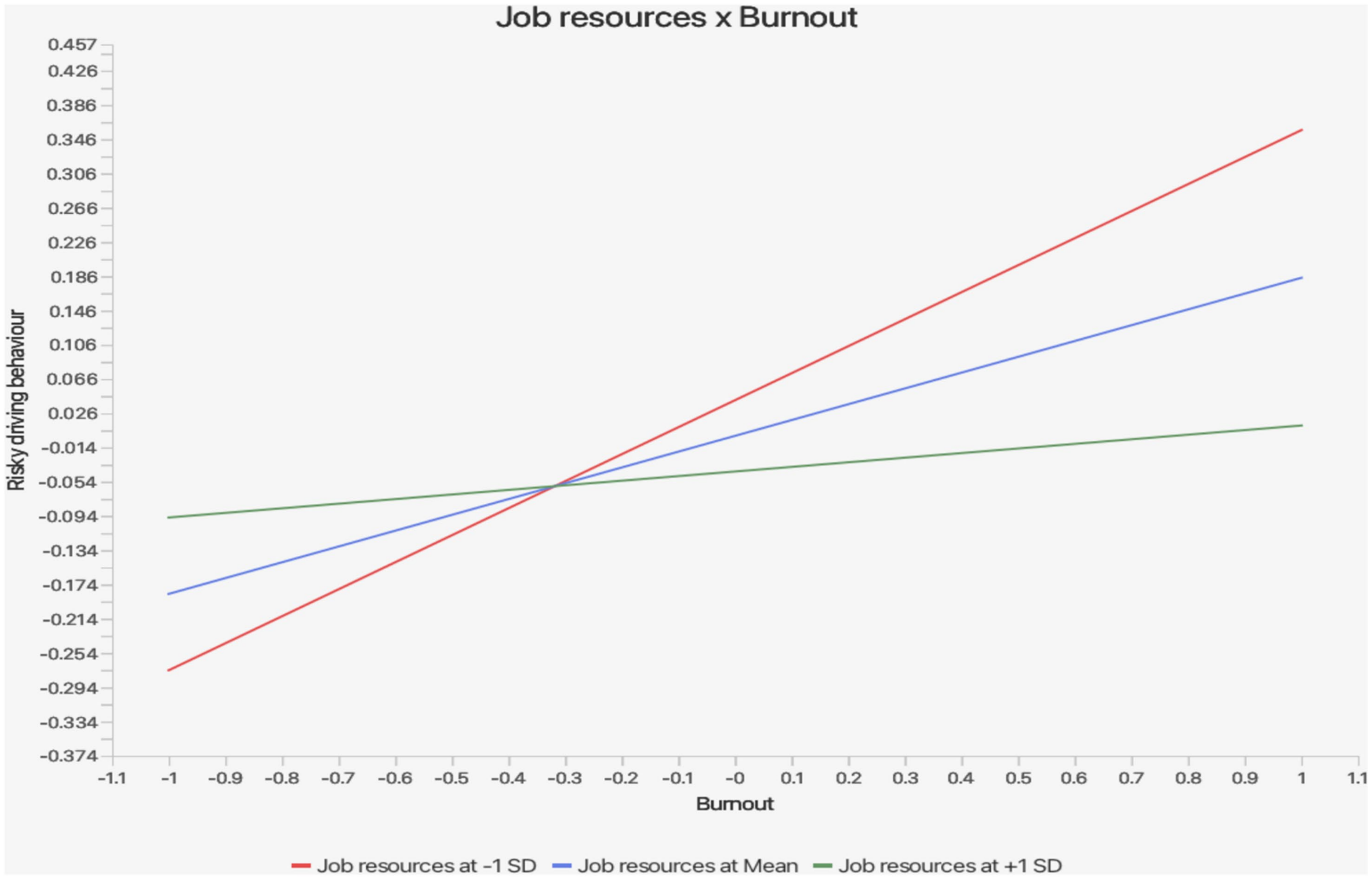
Slope plots for the moderating role of job resources on the path job burnout → risky driving behaviours.

Finally, Hypotheses 12 and 13 were confirmed, showing that job resources moderated the associations between job demand and job burnout, and between job burnout and risky driving behaviours, with moderate effect sizes (*f*^2^ = 0.031 and 0.046; see Table 5). The slope plots for Hypothesis 12 (job resource × job demand → job burnout) are presented in Figure 3, which showed that at low levels of job resource, job demand, and job burnout rose sharply (red line), whereas at high levels of job resource the slope was weak (green line), indicating that job resource may lower the impact of job demand on job burnout. The slope plots for Hypothesis 13 (job resource × job burnout → risky driving behaviours) are shown in Figures 4, 5, indicating that at low levels of job resource, job burnout, and risky driving behaviours rose strongly, but at high levels of job resource the slope flattened (green line), showing that job resource weakens the association between job burnout and risky driving behaviours.

## Discussion

### Summary of findings

The findings confirm that high job demand has a positive association with job burnout and risky driving behaviours among HGV drivers in Ghana. Job burnout has a positive association with risky driving behaviours and is negatively associated with job engagement. High job resources are negatively associated with burnout and positively associated with engagement. However, high job resources did not directly significantly lower risky driving behaviours. Mediation analyses revealed that job burnout partially reduced the effects of job demand on risky driving behaviours, and job resources on risky driving behaviours, while job engagement mediated the relationship between job resources and risky driving behaviours. Moderation analyses showed that job resources significantly buffer the effects of job demand on job burnout, and job burnout on risky driving behaviours.

### Discussion of findings

The study confirms that job demand is associated with both job burnout and risky driving behaviours among HGV drivers in Ghana. This is consistent with the health erosion hypothesis of the JD-R model. High job demands such as long driving hours, tight delivery deadlines, and limited rest are prevalent among Ghanaian HGV drivers, as found in the current study and in the previous ones (17). Job burnout among drivers in this context arises from chronic exposure to these driving demands. The influence of job demand on job burnout and risky driving behaviours is also reported in studies in China (26) and the United States of America (25), reporting that job demand elevates stress and worker job burnout, thereby impairing decision-making, which increases risky behaviours among drivers. Ghana’s unique road transport challenges, such as poor road infrastructure, long distances between rest stops, and inadequate enforcement of rest breaks (17), likely amplified these effects. The significant association between job demand and risky driving behaviours indicates that specific work-related demands, such as extended driving hours and strict delivery timelines, are associated with greater job engagement in unsafe practices such as speeding or driving under fatigue (26). This means that risky driving is not simply individual lapses but may arise from systemic issues within the work environment or poor job design that require urgent attention.

The mediation analysis reveals that job burnout partially mediates the relationship between job demand and risky driving behaviours. This means that while job demand may increase risky driving behaviours, a significant portion of this effect operates through job burnout. Job burnout impairs cognition, including reduced concentration and judgement, and physical functioning ability, which are critical for safe driving (21). Previous studies conducted among professional drivers in China reported similar findings (27, 28), highlighting the role of job burnout in fostering unsafe driving practices. In the Ghanaian setting, the impact of job burnout on risky driving behaviours may be severe due to the absence of appropriate occupational health support systems for these drivers (14). Unlike drivers who benefit from mental health programmes and stress management resources in developed countries such as Spain (10), Ghanaian drivers often lack these job resources or safeguards. However, in the Ghanaian context, peer support and informal check-ins could serve as a culturally relevant and cost-effective approach to alleviating driver job burnout, especially where resource constraints limit formal mental health infrastructure and provision (14). Such grassroots-level strategies may help reinforce coping mechanisms and build resilience, ultimately reducing the risk of unsafe driving behaviours.

The study suggests that high job resources may help lower job burnout and enhance job engagement among the drivers. This supports the motivational hypothesis of the JD-R model, which argues that job resource buffers the effects of job demand by fostering the motivation and resilience of workers (18, 19). Previous studies conducted in China (23) and Colombia (21) among professional drivers corroborate these findings, showing that supportive supervisors and colleagues mitigate stress and promote positive workplace attitudes. Also, among cross-sectoral workers in Belgium, job resources were found to reduce job burnout while improving job engagement (50). In the transport sector in Ghana, however, the effectiveness of job resources may be constrained by systemic limitations. For instance, while support from supervisors could alleviate some stress, structural issues such as inadequate wages and lack of proper training might limit the overall impact of these job resources (17). The enhancement of job engagement through job resources also highlights that engaged drivers are less likely to succumb to job burnout and its associated risks (23). In a developing country context where access to formal mental health services is limited in the transport sector (14), these informal yet contextually relevant support mechanisms could serve as a cost-effective means to bolster job engagement and prevent job burnout.

Interestingly, the findings reveal that job resources did not have a statistically significant association with risky driving behaviours. This is contrary to evidence reported in previous studies (26, 51). This finding also diverges from a prior study (21) that suggests that job resources could directly improve safety outcomes. While job resources improve job engagement and reduce job burnout among the drivers, these effects may not translate into safer driving behaviours (21). The lack of direct impact of this relation among HGV drivers in Ghana may stem from contextual factors such as external pressures on drivers, including unrealistic delivery schedules and financial insecurities. These pressures may overshadow the protective benefits of job resources, forcing drivers to prioritise how to meet the requirements of the job over safety despite supportive environments, from immediate supervisors and co-workers (17). Also, the absence of a direct impact of job resources on risky driving reflects a mismatch between the type of support provided and the practical needs of the drivers. In high-risk environments like HGV driving, social support alone may be insufficient to change on-the-road safety behaviours without the broader systemic supports such as financial incentives or safer infrastructure. Additionally, drivers may normalise risk-taking as part of the job, limiting the behavioural influence of supportive relationships. Moreover, environmental challenges like poor road conditions and weak enforcement of traffic regulations could diminish the influence of job resources on risky driving behaviours among these drivers (17).

The findings show that job engagement plays a significant mediating role in the relationship between job resources and risky driving behaviours. Although the direct effect of job resources on risky driving behaviours was not statistically significant, the indirect pathway through job engagement was significant. This suggests that the influence of job resources operates primarily by strengthening HGV drivers’ psychological connection to their work (35). Thus, simply providing job resources may not automatically reduce risky driving. Instead, these job resources must first enhance drivers’ sense of energy, dedication, and focus before behavioural improvements can manifest or be observed in improved safety in driving.

However, this mediating pathway must be interpreted within the contextual realities of HGV operations in Ghana. Many HGV drivers in Ghana have limited autonomy over trip schedules, rest periods, and delivery timelines, which are often dictated by transport owners, clients or market pressures. Under such structural or systemic conditions, the HGV drivers may find it difficult to fully translate job engagement into safer driving practices, particularly when financial incentives prioritise speed and trip frequency over strict safety compliance (17). This suggests that while job engagement functions as a significant mediator statistically, its practical influence may be partially constrained by systemic and regulatory limitations within the sector.

Nonetheless, the indirect pathway remains theoretically and practically important. Engaged drivers are more likely to exhibit proactive safety behaviours, maintain sustained attention during long-distance travel, and adhere to traffic regulations, while being less inclined to engage in speeding or dangerous overtaking practices (38). This finding aligns with prior evidence demonstrating that work job engagement enhances vigour, concentration, and task absorption, thereby reducing the likelihood of errors and unsafe acts among workers in high-risk occupations (37). In safety-critical roles such as commercial driving, heightened cognitive focus and psychological investment in one’s duties are essential determinants of behavioural compliance.

Furthermore, the results highlight the strategic value of investing in job resources to enhance psychological engagement. Empirical literature suggests that supportive supervision, recognizing workers, fair workload distribution, and adequate rest opportunities strengthen job engagement and performance outcomes (38). Even in the Ghanaian road transport environment characterised by weak enforcement mechanisms, inadequate rest infrastructure, and demanding operational pressures, fostering job engagement may serve as a protective psychological mechanism against risky driving tendencies. Thus, improving job engagement should not be viewed merely as a motivational intervention but also as a practical risk-management strategy capable of indirectly promoting safer driving behaviours among HGV drivers (41).

Moderation analysis shows that job resources significantly buffer the effects of job demand on job burnout, supporting the buffering hypothesis within the JD-R framework (18, 19). Drivers with high job resource experience less job burnout even when facing high job demand. This finding is consistent with the work of Danudoro et al. (44), who highlighted the protective role of job resources in reducing worker stress, suggesting a similar pattern across different occupational contexts. In Ghana, this buffering effect is critical, as it highlights the potential of social support to mitigate the harmful impacts of excessive job demand on these drivers. This finding not only reinforces the buffering hypothesis of the JD-R model but also suggests that even within Ghana’s resource-constrained and informal road transport sector, relational job resources, including supervisor and co-worker support, retain protective value. However, the magnitude of this buffering effect should be interpreted within the structural realities of the sector, where informal employment arrangements and productivity-driven incentives may limit the sustained availability and impact of such a job resource. The moderation analysis further confirms that job resources reduce the impact of job burnout on risky driving behaviours. Drivers with access to job resources are less likely to exhibit unsafe driving behaviours even when experiencing job burnout. This finding is consistent with studies like that of Xanthopoulou et al. (45), which showed that job resources enhanced resilience, enabling workers to maintain performance under strain conditions. This finding is relevant in the Ghanaian road transport sector, as drivers often face chronic job burnout due to high job demand and well-documented systemic challenges (17). While social support from supervisors and colleagues alone cannot eliminate the risks associated with worker job burnout, they provide critical support that helps drivers to cope, potentially reducing the frequency and severity of unsafe driving practices.

Furthermore, the current findings offer theoretical insight by demonstrating the dual moderating role of job resources in both the health erosion and motivation pathways, thereby extending the JD-R model’s applicability in an informal road transport sector in a developing country context. The inclusion of downstream social support as a moderator enriches the model by illustrating how context-specific job resources could shape outcomes under extreme job strain, warranting further testing across diverse occupational groups. However, the JD-R framework assumes that organisations have sufficient structural control to adjust job demand and enhance job resources in a systematic manner. However, in the Ghanaian HGV context, many drivers operate under fragmented ownership structures, informal contractual arrangements, and market-driven payment systems that prioritise trip frequency and associated speed over regulated rest and safety compliance. Under such conditions, job demand may be shaped less by organisational design and more broadly by economic and road infrastructural dynamics. This weakens the extent to which traditional organisational job resources can fully counteract strain, suggesting that the JD-R model may require contextual adaptation when applied to informal sectors. Thus, macro-level factors such as regulatory enforcement, road infrastructure, and economic insecurity appear to function as upstream determinants that influence both job demand and the effectiveness of job resources, thereby extending the model beyond its conventional organisational boundaries.

The findings suggest that job demand and job burnout are the most influential factors associated with risky driving behaviours among the HGV drivers sampled. Higher time pressure, extended driving hours, tight delivery schedules, and performance-based incentives predominant in the Ghanaian road transport industry appear closely linked with elevated strain, which corresponds with greater tendencies towards fatigue-related lapses and risky driving behaviours. Although job resources and job engagement show protective associations, their influence appears largely indirect and comparatively weaker. These patterns indicate the essence of improving structural working conditions, such as regulated driving hours, adequate rest opportunities, and safety-oriented incentive systems, alongside organisational efforts to strengthen driver support and job engagement within the Ghanaian road transport sector.

### Implications for practice, policy, and research

The findings highlight the need for a contextual approach to addressing the psychosocial challenges faced by HGV drivers in Ghana. Reducing job demand through realistic driving schedules and mandatory rest periods is essential to mitigate job burnout and reduce risky driving behaviours. Enhancing job resources, including supervisory support, training, and co-worker collaboration, could alleviate driver stress and promote job engagement. These efforts need to be complemented by systemic changes like improved wages, better road infrastructure, integrating modern OHS standards into the transport sector, and the enforcement of safety regulations. Furthermore, recognising the role of job burnout and job engagement as mediators explains the importance of mental health support that fosters a positive work environment to improve driving safety outcomes. Also, the buffering effect of job resources demonstrates their potential to shield drivers from the adverse impacts of high job demand and job burnout, necessitating targeted efforts to strengthen social and organisational support systems within Ghana’s road transport sector. The inclusion of psychosocial factors such as job burnout and job engagement in road safety frameworks signals a shift from traditional behavioural approaches towards more systemic and worker-centred approaches.

In addition to practical relevance, the study contributes to theoretical advancement by extending the JD-R model in the context of professional driving in a low-resource setting. The study identifies job resources, specifically social support, as a moderating factor that buffers the effects of job demand and job burnout on risky driving behaviours. This highlights the dynamic role of lower-level, downstream job resources in both the health erosion and motivational processes within the JD-R framework. Specifically, support from supervisors and co-workers emerged as critical protective factors, reinforcing the need to priorities such job resources in resource-limited settings where structural support may be lacking or largely inadequate. This provides empirical support for the flexibility of the JD-R model and indicates its applicability to high-risk, under-researched occupational groups, such as HGV drivers in sub-Saharan Africa.

Longitudinal research designs are recommended to establish causal relationships among job demand, job resources, job burnout, job engagement, and risky driving behaviours. Future studies could also explore additional psychosocial and organisational variables, such as job insecurity, emotional labour, and safety climate and heat-stress variables, which may interact with or moderate the effects observed in this model. Moreover, qualitative or mixed-method approaches could provide a deeper understanding of how drivers interpret and navigate psychosocial stressors in their daily work. Comparative studies across different transport corridors or countries in sub-Saharan Africa would further enhance generalisability and reveal context-specific variations in work conditions and safety outcomes. The aim is to build a safer road transport system that protects road users, property, and the economy from the heavy losses caused by traffic accidents.

### Limitations in this study

This study adopted a cross-sectional design, which limits the ability to infer causality among job demand, job resource, job burnout, job engagement, and risky driving behaviours. Additionally, the assessment of risky driving behaviours relied on self-reported data, which could introduce response bias. Participants might have underreported unsafe driving practices or RTCs to present themselves in a favourable light, thereby affecting the accuracy of our findings. Hence, future studies should consider complementing self-reports with objective measures such as GPS tracking data, telematics, or official traffic violation records to improve the validity of risky driving behaviours assessment. Moreover, the sample included only HGV drivers from Tema, Ghana, which may limit how well the findings apply to drivers in other parts of the country or in places with different socio-economic conditions or transport regulations. To enhance external validity, future studies should consider incorporating a broader and more diverse sample of drivers across multiple geographic locations and transport corridors. Such efforts would improve generalisation and provide a comprehensive understanding of how contextual variations influence the relationship between psychosocial work factors and risky driving behaviours.

While the study examined psychosocial factors, it did not account for external variables such as road quality, traffic density, or vehicle maintenance, which may also influence risky driving behaviours. Also, the model did not control for factors such as demographic characteristics, driving experience, crash history, and travel patterns, which may also influence risky driving behaviours. The omission of these variables may limit the comprehensiveness of the model and introduce potential omitted variable bias. Moreover, risky driving behaviours were measured using only five observed indicators, which may not fully capture the complex and multidimensional nature of drivers’ actual on-the-road behaviour. Although the measures demonstrated acceptable psychometric properties, the construct may have been operationalised somewhat narrowly. Hence, future studies should incorporate broader behavioural measures or objective driving data to enhance validity.

## Conclusion

The findings from the current study show that psychosocial work factors are associated with the well-being and safety behaviours of HGV drivers in Ghana. Thus, the data suggest that high job demand was associated with high job burnout and risky driving. Also, the data suggest that high job resources, particularly social support from supervisors and colleagues, were associated with low job burnout and high job engagement. Job burnout and job engagement mediated these relationships, indicating their role in shaping driver outcomes. Furthermore, the data suggest that job resources buffered the effects of job demand and job burnout, highlighting their importance in the work context of HGV drivers in Ghana. These results point to a complex interaction among job demand, job resources, and individual experiences that contribute to understanding driver well-being and on-the-road safety in Ghana. The authors recommend that measures such as ensuring adequate rest periods, setting realistic delivery schedules, and providing supportive work conditions may help reduce job burnout and safety risks among HGV drivers in Ghana.

## Data Availability

The datasets presented in this study can be found in online repositories. The names of the repository/repositories and accession number(s) can be found at: DOI 10.17605/OSF.IO/GF6E3.
